# Differences in physical and mental health symptoms among residents living near municipal solid waste sites: a cross sectional study in the Ashanti Region, Ghana

**DOI:** 10.1186/s41043-024-00527-1

**Published:** 2024-02-29

**Authors:** Prince Peprah, Williams Agyemang-Duah, Anthony Kwame Morgan, Ellen Onyina, Evelyn Serwaa Asare

**Affiliations:** 1 Social Policy Research Centre, University of New South Wales, Sydney, Australia; 2 Centre for Primary Health Care and Equity, University of New South Wales, Sydney, Australia; 3 Department of Geography and Planning, Queen’s University, Kingston, Canada; 4 Department of Geography and Rural Development, Kwame Nkrumah University of Science and Technology, Kumasi, Ghana; 5 Department of Planning, Kwame Nkrumah University of Science and Technology, Kumasi, Ghana

**Keywords:** Municipal solid waste, Landfill, Toxic exposure, Health symptoms, Ashanti region, Ghana

## Abstract

**Objective:**

Physical and mental health concerns and symptoms, including sleep problems, low mood, extreme tiredness, and appetite loss are prevalent among people living near waste sites. This research examines differences in health symptoms among residents living near municipal solid waste sites in the Ashanti Region, Ghana.

**Methods:**

The study used cross-sectional data from 827 residents living near three municipal waste sites, including Besease, Asokore, and Dompoase sites in the Ashanti Region, Ghana. Descriptive statistics, Pearson’s chi-square, and binary logistic regressions were performed to examine the differences and associations between the variables.

**Results:**

Health symptoms, including sleep problems/insomnia, frequent extreme tiredness, low mood, loss of appetite, stress, anxiety, and depression, were reported by the majority of the participants. Residents near open dumpsites (Besease and Asokore) exhibit significantly higher likelihoods of experiencing various health symptoms such as extreme fatigue, depression, psychological disorders, thinking and concentration problems, low mood, loss of appetite, and anxiety compared to those near the engineered Oti landfill in Dompoase.

**Conclusion:**

While emphasizing the importance of proper landfill design and management in Ghana, this study underscores the need for further longitudinal and clinical investigations. Clinically establishing the link between dumpsites and health symptoms is imperative for informed public health interventions and policy decisions aimed at mitigating the potential adverse health effects of landfills on residents' well-being.

## Introduction

Globally, approximately 1.3 billion tons of solid waste are generated annually, and the volume is projected to reach 2.2 billion tons annually by 2025 [16, 45]. High rates of urbanisation, low recycling, and reuse rates, as well as a shift in consumption patterns, have resulted in an increase in waste generation in many regions, including sub-Saharan Africa (SSA) [5, 16]. In SSA, the management of large volumes of waste, including collecting, transporting, and processing solid waste amid the high rate of urbanisation, is challenging for most countries within the region [25].

Ghana’s population is increasing rapidly, without any accompanying increase in sanitation and infrastructure development. Ghana's sanitation coverage was reported to be 21% in 2018, and despite a population exceeding 30 million in 2021, only 25% have access to sanitation services, highlighting a disparity in infrastructure development [13, 14, 47]. This suggests a significant gap between rapid population growth and insufficient progress in sanitation and infrastructure development.

This trend places great stress on waste management facilities in the country [37]. The most common waste disposal option in Ghana involves the collection and dumping of mixed materials at the chosen sites. These sites are open spaces in and on the outskirts of towns [7]. The indiscriminate disposal of waste in and around the cities in Ghana has contributed to the occurrence of wastelands and dumpsites. Waste management facilities in Ghana focus largely on waste collection, while neglecting waste management 30. This limited access to sanitation services poses considerable challenges to public health and underscores the pressing need for concerted efforts to improve the sanitation infrastructure and services across the country.

Research and anecdotal evidence suggest that municipal solid waste landfills remain the most preferred and commonly used solid waste disposal option in Ghana. This is mainly due to its perceived cost-effectiveness and the absence of more innovative waste management technologies [1, 39, 40]. However, solid waste disposal at landfill sites has raised concerns about possible adverse health effects on nearby people [29, 41, 50]. In many instances, poor landfilling is regarded as the opposite of sustainable development owing to associated health and environmental constraints 36.

The health risks associated with proximity to poorly managed landfills are diverse and extensive. For instance, previous studies have revealed that residents living close to landfill sites suffer from many health conditions, such as asthma, eye problems, reproductive diseases, and many others, more than people who live far away from those sites [3, 6, 7, 18, 31, 32, 41, 43]. Health disorders such as cancer, mortality, and other reproductive issues have also been clinically and reportedly linked to landfills in some areas in developed countries [21, 51] and developing countries [4, 37, 39–41]. Other toxic exposure symptoms such as irritation of the eye, throat, and nose, headaches, fatigue, and running nose have been reported [4, 20, 23, 32, 35]. Residents in Lagos State, Nigeria, living near landfill sites, reported higher occurrences of respiratory and skin disorders, including wheezing, frequent sneezing, unpleasant odours, fever, and skin rashes, than those residing farther away [2]. Singh et al. [45] discovered that increased exposure to the dumping site in Mumbai, India, resulted in a higher prevalence of respiratory illness (12%), eye irritation (8%), and stomach problems (7%). Multivariate analysis indicated that the respondents from the exposed group were significantly more likely to experience respiratory illnesses, eye infections, and stomach problems. A systematic review by Vinti et al. [49] identified an elevated risk of mortality, respiratory diseases, and adverse mental health effects in individuals living near landfills. The study also provided some evidence of an increased mortality risk associated with residing near incinerators.

However, the literature on the health impact of landfill sites is inconclusive, with little evidence of the differences in health symptoms among residents living near landfill sites, especially in the context of developing countries, specifically Ghana. The only study that specifically examined the differences in health symptoms among residents living near dumpsites was conducted in Mexico [4]. This study in Mexico [4] focused on illegal dumpsites and did not examine health symptoms among residents near illegal and legal and/or open and engineered landfill sites. To the best of our knowledge, no study has specifically assessed differences in self-reported health symptoms among residents living close to landfills in Ghana, which has created a knowledge gap in the landfill and health literature. For instance, Peprah et al. [41] examined the prevalence and comorbid factors of ocular allergies among residents living near the Dompoase landfill site in Kumasi, Ghana. However, aside from studying a single landfill site, their research did not examine differences in the health conditions studied. It is therefore imperative to examine the differences in health symptoms among residents living near open and engineered municipal solid waste sites in Ghana to offer evidence to aid in policy formulation on the strategies that aim to curb the impacts of landfills on their health.

## Methods

### Study design

This was a descriptive epidemiological study with a cross-sectional approach. We adopted this approach because it provides a conducive environment for assessing differences in health symptoms among residents living near dumpsites. It also offers good grounds for the development of hypotheses that can be tested later by more powered and longitudinal studies [15, 24, 50].

### Study setting

The study region was the Ashanti Region, which was purposively selected based on recent frequent public adverse reactions toward landfills in the region [33], Owusu-Sekyere et al. 40. Specific landfill site communities within the region were selected through simple random probability selection without replacement, as one of the researchers was blindfolded to pick from a pool of purposive lists of landfill/waste disposal sites known to the researchers and other stakeholders. Three (3) landfill site communities were selected: Ejisu-Besease in Ejisu-Juaben Municipal, Asokore in Sekyere East District, and Oti in Asokwa Municipal. The landfill sites have different characteristics. For instance, it is worth noting that of these three landfills, only the Oti landfill is an engineered landfill, with the remaining two being unsanitary. The Oti landfill is the only sanitary landfill in the Ashanti Region, serving approximately 1.7 million inhabitants in Kumasi, the largest city after Accra [39]. The remaining two landfills serve the waste disposal needs of the people in Ejisu, Effiduase, and the adjoining towns. Existing empirical and anecdotal evidence indicate that the operation and management of dumpsites, including the Oti landfill which is an engineered landfill, has remained stable [38, 40]. These sites mostly lack basic facilities and equipment, such as fences, liners, soil cover, and compactors, and are located close to wetlands, water sources, and communities.

### Subject and selection

A cohort of residents within a 2 km zone of the Oti landfill site, Asokore, and Besease dumpsites were enrolled in the study. In the absence of locational accuracy, the 2 km distance within each site is in line with previously established observations and practices [41]. A 2 km zone around each site was constructed using Geographic Information System (GIS) techniques. Given the estimated proportion of households that reside in the 2 km zone of the sites, a sample size of 926 individuals was drawn from the population of interest. Specifically, we calculated the sample for each site using the Lwanga and Lemeshow [22] formula, n = [(Zα/2)2 × P (P − 1)]/ε2 for sample size calculation for health research with a margin of error of 0.05, confidence interval of 95% (Zα/2 = 1.96) as well as proportion of 0.40, 0.175 and 0.168 for Dompoase, Asokore and Besease, respectively. By inserting the parameters into the above formula, minimum sample sizes of 369, 222, and 215 for Dompoase, Asokore and Besease, respectively, were determined. Considering a 10.5% non-response rate, the final sample size for each site was approximated to 409, 262, and 255 participants for Dompoase, Asokore, and Besease, respectively.

Systematic random sampling was used to ensure fair representation and precision [11]. Every 5th household was sampled and surveyed until the required number of respondents were obtained, with a response based on recommendations from Cooper, Schindler, and Sun [8]. To be included in the study, a participant must be: (1) living in the designated 2 km zone of the landfill area; (2) a household head and; (3) 18 years or above.

### Data collection

This study used a questionnaire developed in English for data collection. The questionnaire was developed by the researchers based on a literature review. We administered the questionnaires using a face-to-face interviewer-administered approach to increase the response rate due to the inability of most respondents to read and write in English. The questionnaire included questions about ten perceived toxic exposure symptom variables among households completing the questionnaire. Although the questions were formulated in English, they were translated into Twi (the local language of the participants), taking into consideration the World Health Organization guidelines for assessment of instruments [48]. The translation was performed by the first author, followed by independent checks and re-checking by the authors to ensure quality control. Fifteen (15) trained field assistants collected data using a door-to-door approach from 10 June to 30, 2019. Before data collection, piloting and testing of the questionnaire were conducted with 29 respondents who did not form part of the main sample but shared similar characteristics. This pilot enhanced the validity and reliability of the questions through revisions made based on feedback from the piloting. The field assistants constituted people with research experience and hail from the study communities, and could speak the local dialect fluently. As participants may not understand these clinical terms, field enumerators explained these conditions in the participants’ local language. The interviewers explained to each participant that the purpose of the study was to assess their perceived health symptoms, and written informed consent was obtained. The residents were unaware that their responses would be analysed within the context of adjacency to dumpsites and were unaware of the possible link between these symptoms and environmental exposure. The first author monitored the data collection process to ensure that field assistants adhered to appropriate data collection principles in the field. The choice not to inform participants about the specific focus on adjacency to dumpsites and the potential link between symptoms and environmental exposure raise ethical concerns, suggesting a lack of transparency and potential deception. Nonetheless, the decision was rooted in preventing potential biases or overestimation of health effects that could arise if participants were aware of the study's specific focus. While transparency is crucial, we argue that this approach was necessary to obtain more objective and unbiased responses regarding health symptoms, minimising the risk of participants attributing symptoms solely to their proximity to the dumpsites.

### Measures

The dependent variable in this study was health symptoms. Health symptoms were considered in this study, based on previous findings [4, 20, 23, 42, 44]. These symptoms included (1) frequent extreme tiredness (fatigue); (2) psychological disorders; (3) depression; (4) low mood; (5) anxiety; (6) loss of appetite; (7) thinking and concentration problems; (8) stress; (9) eye irritation; and (10) sleep problems/insomnia. Respondents were asked to answer yes or no for each perceived health symptom. In this study, psychological disorders were viewed as a comprehensive category encompassing abnormal thoughts, emotions, or behaviours that significantly impair functioning. Within this umbrella term, specific manifestations include depression, which is identified as a distinct disorder characterised by persistent feelings of sadness and a lack of interest in daily activities. The term "low mood" is employed as a general descriptor, indicating a temporary and mild decrease in the emotional state. Additionally, "anxiety" is employed to characterise excessive worry, fear, or nervousness. These terms, treated as unique dimensions within the broader category of psychological disorders, provide a nuanced understanding of mental health challenges experienced by individuals living near landfills.

The independent variable in this study was place of residence. This variable was measured by a single question asking respondents to select their place of residence from one of the study locations, including Dompoase, Asokore, and Besease. Consistent with the results of previous studies [4, 42], the analysis was adjusted for various variables including age, sex, education, length of stay in the community, education, employment status, and monthly income.

### Data analysis

A total of 926 respondents were recruited; 827 participants fully completed the questionnaire, yielding a response rate of 89.3%. All statistical analyses were performed using SPSS software version 27.0 (SPSS Inc., Chicago, IL). Descriptive analysis using percentages and frequencies was performed to describe and contextualise the sample. Pearson’s chi-square tests were applied and all the variables with  a *p*-value < 0.05 were selected for binary logistic regression and calculation of odds ratios to further examine the relationships between place of residence and each of the ten dichotomised perceived health symptoms. A 95% confidence interval was used to determine statistical significance.

### Ethics approval and consent to participate

The Committee on Human Research Publication and Ethics (CHRPE), School of Medical Sciences, Kwame Nkrumah University of Science and Technology, and Komfo Anokye Teaching Hospital, Kumasi, Ghana, provided ethical clearance for this study. Informed written and verbal consent was obtained from the study participants before data were collected. The study procedures and protocol were conducted according to the tenets of the Declaration of Helsinki. The study participants were also assured of the strict confidentiality and anonymity of the data they provided.

## Results

### Demographic and socio-economic characteristics of residents living near landfill sites by place of residence

The demographic and socioeconomic characteristics of the residents living near landfill sites by place of residence are shown in Table 1. The results showed that 52.4% of the participants were female, 54.1% were between 30 and 39 years of age, 40.7% had stayed in the community for less than 5 years, 42.7% had completed basic school education, 73.6% were employed, 35.4% earned a monthly income of 101–300 cedis, 54.1% sourced water from treated sources, 80.2% had registered for health insurance, and 84.8% rated their health status as good or very good. The results further highlighted a statistically significant difference between gender, age, length of stay in the community, employment status, monthly income, and source of water in relation to place of residence (see Table 1).

**Table 1:** Demographic characteristics of residents living near the landfill sites

Variable	Categories	Community				
		Asokore (218)	Besease (209)	Dompoase (400)	Total (827)	p-Value
Gender	Male	156 (71.6)	36 (17.2)	202 (50.5)	394 (47.6)	0.000*
	Female	62 (28.4)	173 (82.8)	198 (49.5)	433 (52.4)	
Age (years)	18–29	75 (34.4)	65 (31.1)	126 (31.5)	266 (32.2)	0.010^*^
	30–39	103 (47.2)	107 (51.2)	237 (59.2)	447 (54.10)	
	40–49	22 (10.1)	18 (8.6)	18 (4.5)	58 (7.0)	
	50 and above	18 (8.3)	19 (9.1)	19 (4.8)	56 (6.8)	
Length of stay in the community	< 5 yrs	127 (58.3)	82 (39.2)	128 (32.0)	337 (40.7)	< 0.001^*^
	5–10yrs	69 (31.7)	68 (32.5)	172 (43.0)	309 (37.4)	
	11–15yrs	14 (6.4)	14 (6.7)	82 (20.5)	110 (13.3)	
	16–20yrs	7 (3.2)	11 (5.3)	16 (4.0)	34 (4.1)	
	All my life	1 (0.5)	34 (16.3)	2 (0.5)	37 (4.5)	
Education	No formal education	30 (13.8)	31 (14.8)	66 (16.5)	127 (15.4)	0.086
	Basic school education	92 (42.2)	107 (51.2)	154 (38.5)	353 (42.7)	
	High school education	74 (33.9)	50 (23.9)	135 (33.8)	259 (31.3)	
	College/tertiary	22 (10.1)	21 (10.0)	45 (11.2)	88 (10.6)	
Employment status	Not employed	47 (21.6)	45 (21.5)	126 (31.5)	218 (26.4)	0.005^*^
	Employed	171 (78.4)	164 (78.5)	274 (68.5)	609 (73.6)	
Monthly income (GH¢)	Less or equal to 100.00	16 (7.3)	37 (17.7)	24 (6.0)	77 (9.3)	< 0.001^*^
	101.00–300.00	67 (30.7)	63 (30.1)	163 (40.8)	293 (35.4)	
	301.00–500.00	54 (24.8)	35 (16.7)	88 (22.0)	177 (21.4)	
	501.00–700.00	41 (18.8)	20 (9.6)	52 (13.0)	113 (13.7)	
	More than 700	30 (13.8)	49 (23.4)	61 (15.2)	140 (16.9)	
Source of water	Treated sources	117 (53.7)	125 (59.80	205 (51.2)	447 (54.1)	0.131
	Non-treated sources	101 (46.3)	84 (40.2)	195 (48.8)	380 (45.9)	

*The Chi-square statistic is significant at the 0.05 level

### Health status or behavior of residents living near the landfill sites

Table 2 presents the results across the three communities—Asokore, Besease, and Dompoase. The results indicated that approximately 80.2% of the respondents were enrolled in health insurance (NHIS), with no notable variation observed across the communities (*p* = 0.804). Also, a significant difference was found in self-rated health status, with 39.0% in Asokore rating their health as "very good”, compared to 35.4% in Besease and 42.0% in Dompoase (*p* = 0.038*). Approximately 30.4% of respondents reported alcohol consumption in the past year, showing no significant difference between communities (*p* = 0.057). Notably, a significant discrepancy in smoking habits emerged across communities (*p* = 0.001*), revealing that 6.4% of Asokore respondents smoked in the past year, compared to 1.0% in Besease and 13.6% in Dompoase.

**Table 2:** Health status and behavior of residents living near the landfill sites

Variable	Categories	Community				
		Asokore (218)	Besease (209)	Dompoase (400)	Total (827)	p-Value
Have you ever registered for health insurance (National Health Insurance Scheme)?	Yes	176 (80.7)	170 (81.3)	317 (79.2)	663 (80.2)	0.804
	No	42 (19.3)	39 (18.7)	83 (20.8)	164 (19.8)	
In general, how would you rate your health today?	Very good	85 (39.0)	74 (35.4)	168 (42.0)	327 (39.5)	0.038*
	Good	111 (50.9)	98 (46.9)	166 (41.5)	375 (45.3)	
	Fair	17 (7.80	20 (9.6)	33 (8.2)	70 (8.5)	
	Poor/very poor	5 (2.3)	17 (8.1)	33 (8.2)	55 (6.7)	
In the past one year, have you ever consumed alcohol?	Yes	33 (27.0)	27 (22.9)	134 (33.6)	194 (30.4)	0.057
	No	89 (73.0)	91 (77.1)	265 (66.4)	445 (69.6)	
In the past one year, have you smoked before?	Yes	14 (6.4)	2 (1.0)	54 (13.6)	70 (8.5)	0.001*
	No	204 (93.6)	207 (99.0)	343 (86.4)	754 (91.5)	

*The Chi-square statistic is significant at the 0.05 level

### Perceived health symptoms among residents living near a landfill site by place of residence

The results of the respondents’ perceptions of health symptoms by residence are shown in Table 3. The study revealed that 62% of participants reported frequent extreme tiredness, 50.5% reported psychological disorders, 57.4% reported depression, 58.6% experienced low mood, 57.7% suffered from anxiety, 61.2% experienced loss of appetite, 49.1% reported thought and concentrated problems, 57.9% experienced stress, 49.9% reported eye irritation, and 70.7% indicated sleep problems/insomnia. The results further showed a statistically significant association between perceived depression, low mood, anxiety, loss of appetite, thinking and concentrating problems, stress, eye irritation, and sleep problems or insomnia and the community of residence (see Table 3).

**Table 3:** Perceived health symptoms among residents living near the landfill sites

		Community				
		Asokore (218)	Besease (209)	Dompoase (400)	Total (827)	*p*-Value
Frequent extreme tiredness (fatigue)	Yes	119 (54.6)	130 (62.2)	264 (66.0)	513 (62.0)	< 0.001^*^
	No	99 (45.4)	79 (37.8)	136 (34.0)	314 (38.0)	
Psychological disorders	Yes	62 (28.4)	105 (50.2)	251 (62.7)	418 (50.5)	< 0.001^*^
	No	156 (71.6)	104 (49.8)	149 (37.2)	409 (49.5)	
Depression	Yes	88 (40.4)	123 (58.90	264 (66.0)	475 (57.4)	< 0.001^*^
	No	130 (59.6)	86 (41.1)	136 (34.0)	352 (42.6)	
Low mood	Yes	88 (40.4)	131 (62.7)	266 (66.5)	485 (58.6)	< 0.001^*^
	No	130 (59.6)	78 (37.3)	134 (33.5)	342 (41.4)	
Anxiety	Yes	79 (36.2)	123 (58.9)	275 (68.8)	477 (57.7)	< 0.001^*^
	No	139 (63.8)	86 (41.1)	125 (31.2)	350 (42.3)	
Loss of appetite	Yes	90 (41.3)	120 (57.4)	296 (74.0)	506 (61.2)	< 0.001^*^
	No	128 (58.7)	89 (42.6)	104 (26.0)	321 (38.8)	
Thinking and concentrating problems	Yes	70 (32.1)	108 (51.7)	228 (57.0)	406 (49.1)	< 0.001^*^
	No	148 (67.9)	101 (48.3)	172 (43.0)	421 (50.9)	
Stress	Yes	85 (39.0)	131 (62.7)	263 (65.8)	479 (57.9)	< 0.001^*^
	No	133 (61.0)	78 (37.3)	137 (34.2)	348 (42.1)	
Eye irritation	Yes	64 (29.4)	96 (45.9)	253 (63.2)	413 (49.9)	< 0.001^*^
	No	154 (70.6)	113 (54.1)	147 (36.8)	414 (50.1)	
Sleep problems/ insomnia	Yes	113 (51.8)	172 (82.3)	300 (75.0)	585 (70.7)	< 0.001^*^
	No	105 (48.2)	37 (17.7)	100 (25.0)	242 (29.30	

*The Chi-square statistic is significant at the 0.05 level

### Association between place of residence and health symptoms

The results of the association between the health symptoms of respondents and place of residence among residents living near a landfill site are reported in Table 3. In the univariable analysis, the results have shown that respondents residing in Asokore were significantly more likely to report fatigue (COR 1.615, C1:1.152–2.263), depression (COR 2.868, CI 2.040–4.031), psychological disorder (COR 4.239, CI 2.965–6.059), thinking and concentrating problems (COR 2.80, CI 1.982–3.963), low mood (COR 2.932, CI; 2.085–4.124), loss of appetite (COR 4.048, CI 2.852–5.745), anxiety (COR 3.871, CI 2.734–5.481), stress (COR 3.004, CI 2.134–4.227), eye irritation (COR 4.141, CI 4.141) and sleep problems/insomnia (COR 2.788, CI 1.966–3.952) compared to those who were residing at Dompoase. In the same univariable analysis, the study further revealed that respondents living in Besease had significantly higher odds of experiencing psychological disorders (COR 1.669, CI 1.189–2.341), loss of appetite (COR 2.111, CI 1.482–3.007), anxiety (COR 1.538, CI 1.087–2.177)), stress (COR 3.004, CI 2.134–4.227), eye irritation (COR 2.026, CI 1.443–2.845), and sleep problems/insomnia (COR 0.645, CI 0.424–0.983) compared to those who were residing at Dompoase.

After adjusting for theoretically relevant demographic and socio-economic variables in a multivariable analysis, the study revealed that respondents residing in Asokore were significantly more likely to report fatigue (AOR 1.640, CI 1.023–2.631), depression (AOR 3.102, CI 1.885–5.105), psychological disorders (AOR 4.313, CI 2.558–7.273), thinking and concentrating problems (AOR 2.104, CI 1.303–3.399), low mood (AOR 2.528, CI 1.562–4.093), loss of appetite (AOR 4.563, CI 2.787–7.472), anxiety (AOR 4.024, CI 2.451–6.608), stress (AOR 2.763, CI 1.705–4.477), eye irritation (AOR 4.743 CI 2.828–7.953) and sleep problems/insomnia (AOR 2.635, CI 1.586–4.378) compared to those who reside at Dompoase. In addition, participants from Besease were significantly more likely to report depression (AOR 1.898, CI 1.102–3.269), thinking and concentrating problems (AOR 1.814, CI 1.071–3.075), loss of appetite (AOR 3.178, CI1.837–5.500), eye irritation (AOR 2.844 CI 1.666–4.856), and sleep problems/insomnia (AOR 0.446, CI 0.222–0.893) compared to respondents who reside at Dompoase (see Table 4). The take-home message is that participants residing at Besease and Asokore were significantly more likely to report varied health symptoms than those residing at Dompoase.

**Table 4:** Univariable and multivariable logistic regression models

Variables in the equation																				
	Fatigue		Depression		Psychological disorders		Thinking and concentrating problems		Low mood		Loss of appetite		Anxiety		Stress		Eye irritation		Sleep problems/insomnia	
	Unadj	Adj ^a^	Unadj	Adj ^a^	Unadj	Adj ^a^	Unadj	Adj ^a^	Unadj	Adj ^a^	Unadj	Adj ^a^	Unadj	Adj ^a^	Unadj	Adj ^a^	Unadj	Adj ^a^	Unadj	Adj ^a^
Asokore	1.615* (1.152–2.263)	1.640* (1.023–2.631)	2.868* (2.040–4.031)	3.102* (1.885–5.105)	4.239* (2.965–6.059)	4.313* (2.558–7.273)	2.803 (1.982–3.963)	2.104* (1.303–3.399)	2.932* (2.085–4.124)	2.528* (1.562–4.093)	4.048* (2.852–5.745)	4.563* (2.787–7.472)	3.871* (2.734–5.481)	4.024* (2.451–6.608)	3.004* (2.134–4.227)	2.763* (1.705–4.477)	4.141* (2.903–5.908)	4.743 (2.828–7.953)	2.788* (1.966–3.952)	2.635* (1.586–4.378)
Beasease	1.180 (0.833–1.670)	1.269 (0.749–2.150)	1.357 (0.962–1.916)	1.898* (1.102–3.269)	1.669* (1.189–2.341)	1.022 (0.575–1.817)	1.240 (0.886–1.734)	1.814* (1.071–3.075)	1.182 (0.834–1.675)	1.330 (0.773–2.286)	2.111* (1.482–3.007)	3.178* (1.837–5.500)	1.538* (1.087–2.177)	1.331 (0.776–2.284)	1.143 (0.807–1.619)	1.428 (0.839–2.429)	2.026* (1.443–2.845)	2.844* (1.666–4.856)	0.645* (0.424–0.983)	0.446* (0.222–0.893)
Dompoase (Reference Group)																				

Unadj = Unadjusted Odd Ratio: Adj = Adjusted Odd Ratio

*p-value of 0.05 or less

^a^Adjusted for age, sex, education, length of stay in the community, education, employment status and monthly income

## Discussion

In this study, we examined the perceived health symptoms of residents of Ghana's Ashanti region, who live near municipal solid waste sites because of the potential health implications of landfills. The findings revealed that the majority of residents reported various health symptoms, including sleep problems, extreme tiredness, low mood, loss of appetite, stress, anxiety, and depression. Multivariable analysis indicated that residents near open dumpsites in Besease and Asokore were significantly more likely to experience a range of health issues than those residing near the engineered dumpsite in Dompoase (Oti landfill). Noteworthy the health symptoms include extreme fatigue, depression, psychological disorders, thinking and concentration problems, low mood, loss of appetite, and anxiety. This study emphasises the importance of proper landfill design and management in Ghana, while highlighting the need for further longitudinal and clinical investigations to establish a clinical link between dumpsites and health symptoms.

The perceived health symptoms significantly varied across the three study sites, with participants residing at Besease and Asokore being significantly more likely to report varied health symptoms compared to those residing at Dompoase. The marked differences in the exposure to health symptoms by residents living near landfills from the various study sites, with dompoase being the least exposed, could be underpinned by the intensity of exposure at each site and the differences in the management of the various landfills [4, 19]. Focusing on the built-up and makeup of the sites, the Dompoase landfill is engineered/sanitary/modern compared to that of Besease and Asokore which are open dumpsites. As such, the Dompoase landfill has certain conditions and facilities that can reduce its health impacts on the environment, such as seepage services and fences. These modernised landfills are built on the idea of segregating landfills from the environment to properly consolidate waste and render them harmless by biological, chemical, and physical treatments. Landfill management needs to prioritise the design and operation of sustainable landfills. Therefore, it is recommended that more engineered and standard landfills/waste sites be developed in Ghana to reduce the environmental and health consequences associated with unengineered landfills.

In their cross-sectional survey of health symptoms exhibited by residents living near illegal dumpsites in Mexico, Al-Delaimy et al. [4] observed differences in the makeup and proximity of residents across the sites they studied, partly explaining the observed differences in the likelihood of exposure to pollution among residents. Similarly, our study sites were not homogenous because the topography, wind direction, and other environmental factors could intensify the possibility of exposure to hazardous substances from landfills. The number and contents of the dumpsite may also play a role in how residents are exposed to these hazards [28, 34]. This brings into focus the argument of Khoiron et al. 19 that the appropriate management of landfills, in accordance with current environmental legislation and standards, limits their detrimental influence on the environment and public health. The sheer volume of waste sent to the site has made it receive “extreme” attention from policy and research landscapes. This could, in part, explain the relatively better management strategy compared to that of Asokore and Besease, with a lower likelihood of self-reported health symptoms being a consequence.

Communities near landfills and open dumps are vulnerable to the health hazards of exposure to landfill gases 12. Based on our findings, health symptoms of extreme tiredness, psychological disorders, loss of appetite, stress, and depression, in addition to low mood, anxiety, thinking and concentrating problems, eye irritation, and sleep problems/insomnia, were present in the sample. These findings are consistent with earlier research documenting the symptoms that occur when people are exposed to harmful substances in the environment [4, 10, 17, 20, 26, 45]. This research suggests that inappropriate solid waste management might pose health concerns to humans, particularly for those who live near landfills. The inadequacy of information on the health-related impacts of landfills, with available evidence often drawn from self-reported surveys as against bio-monitoring, poses a significant challenge to understanding the concept and variations across space and time [46]. Overall, the existence of a landfill has a detrimental influence on the environment and public health, according to the findings of this study. However, if landfill management is performed correctly and in accordance with the current legislation and standards, the negative effects can be reduced.

Significantly, this study contributes to understanding the nexus between living near landfills and self-reported health symptoms from exposure to hazardous substances. The strength of this study lies in the fact that it is the first large-scale regional-level study to explicitly examine this subject matter. Therefore, our findings are imperative for prompting research into the subject matter in Ghana while providing policy options for development practitioners in the space of waste management. This study further demonstrates that residents living near landfills in Ghana’s Ashanti region suffer from extreme tiredness, psychological disorders, loss of appetite, and stress. Considering this finding, further research is needed to determine which toxicants residents are exposed to and the source of that exposure. The use of biomarkers might help further understand the link between exposure to hazardous substances in the environment and symptoms. Blood, urine, breast milk, and toenails should be used in investigations to identify harmful chemical exposure in the environment.

Recognising the often-prolonged nature of policy formulation in Ghana, it is imperative for immediate, community-driven initiatives. Residents should be empowered with information on waste management practices, emphasising appropriate waste disposal methods, recycling, and community-led cleanliness campaigns. Collaborative efforts among local authorities, environmental agencies, and community leaders are essential for implementing sustainable waste management strategies. Additionally, health awareness programs should be conducted to educate residents about potential health hazards and symptoms related to dumpsite proximity, enabling them to seek timely medical attention. While advocating for efficient waste management policies at the national level remains crucial, these localised actions can contribute to immediate improvement in residents' health outcomes and create a cleaner, healthier living environment.

Despite these strengths, some limitations of this study must be highlighted. This study relied solely on self-reported health effects and perceived risks, which may have led to subjectivity and recollection bias among participants. Given the structure of Ghana's healthcare system, where there is no specific clinic or hospital in the region that the residents visit, confirming the residents' symptoms with health outcome data from local health clinics was not possible. To control this, blood, urine, breast milk, and toenails should be used in investigations to identify harmful chemical exposure in the environment. Again, because cause-and-effect links were not established in this study, other factors might have influenced the reported patterns. Future longitudinal and clinical studies should be conducted to determine and track the effects of exposure to hazardous substances on the health of people living near landfills.

## Conclusion

This study underscores the critical issue of environmental and public health implications for communities residing near landfills in Ghana. Exposure to various air pollutants, contaminated soil, and water near these sites poses serious health risks to residents. The reported symptoms serve as a preliminary indication of potential health consequences, emphasising the need for comprehensive epidemiological evaluation for effective long-term health management. As an urgent recommendation, clear policy directions for the proper management of landfills are imperative to mitigate the environmental and public health consequences experienced by those living in proximity to these sites. To address this challenge, it is essential to focus on the development and reinforcement of institutional arrangements that involve diverse stakeholders. Additionally, awareness campaigns should be initiated to educate communities and policymakers regarding the risks associated with landfill proximity. Moreover, this study advocates the adoption and replication of innovative landfill management technologies as evidence of good governance. Local public agencies, serving as the core of development, must integrate economic opportunities, health considerations, and environmental impact assessments into landfill design and management. This approach will ensure a more holistic and sustainable landfill management strategy, minimising detrimental influences on the environment and public health.

## Data Availability

The datasets used and/or analysed during the current study are available from the corresponding author upon reasonable request.
